# Survey of Reactive Hyperplastic Lesions of the Oral Cavity in Mashhad, Northeast Iran

**DOI:** 10.5681/joddd.2011.029

**Published:** 2011-12-19

**Authors:** Maryam Amirchaghmaghi, Nooshin Mohtasham, Pegah Mosannen Mozafari, Zohreh Dalirsani

**Affiliations:** ^1^Dental Research Center, Mashhad University of Medical Sciences, Mashhad, Iran; ^2^Associate Professor, Department of Oral Medicine, Dental School, Mashhad University of Medical Sciences, Mashhad, Iran; ^3^Associate Professor, Department of Oral and Maxillofacial Pathology, Dental School, Mashhad University of Medical Sciences, Mashhad, Iran; ^4^Assistant Professor, Department of Oral Medicine, Dental School, Mashhad University of Medical Sciences, Mashhad, Iran

**Keywords:** Diagnosis, histopathology, oral mucosa, reactive hyperplasia

## Abstract

**Background and aims:**

Reactive hyperplasias (RHs) are common oral mucosal lesions caused by irritation of the mu-cosa. The aim of this study was to evaluate the frequencies of various types of oral RHs in a university clinic.

**Materials and methods:**

Oral RH cases, undergone biopsy in a four-year period, were studied retrospectively. Data including age and gender as well as the location and clinical characteristics of the lesion were collected and analyzed.

**Results:**

A total of 123 cases (age range 7-79 years old; mean: 38.8 ± 18.50 years; male: female ratio, 1.9:1) were evalu-ated. Over a half of the lesions including pyogenic granuloma, giant cell granuloma, and peripheral ossifying fibroma were found in gingiva (52%). Fibroma involved mostly the buccal mucosa. Most lesions had smooth surface and sessile base.

**Conclusion:**

The clinical features of oral inflammatory hyperplasia in the studied sample were similar to those previously reported.

## Introduction

The oral mucosa is exposed to chronic or recurrent irritations such as calculus, ill fitting dentures and overhanging dental restorations,^1 , 2^ which lead to various reactive hyperplasias (RHs) that histologically represent chronic inflammation, granulation tissue and proliferation of endothelial cells and fibroblasts.^1 , 3^ In some of these lesions, such as pregnancy tumor, the level of circulating hormones play a role.^1 , 4^

These lesions may be large or small in size. These tumor-like lesions indicate a chronic process, in which an exaggerated repair occurs following injury and usually have no radiographic features.^3 , 4^ However, in some cases, erosion and cup-shaped radiolucency occurs in the underlying bone. Clinical appearance consists of sessile or sometimes pedunculated masses with either ulcerated or intact smooth surface. Surgical excision is the treatment of choice in RHs and elimination of chronic irritant is mandatory. The majority of RHs will not recur.^1^ However, if the source of trauma persists, frequent recurrences are possible.

Clinical behavior of RHs may vary in different populations, reflecting different environmental factors, life styles and racial factors. In spite of a considerable literature, limited data are available on RHs in the Iranian population. The purpose of this study was to describe the clinical features of RH cases with a histopathologic diagnosis, treated at a university clinic.

## Materials and Methods

The patient files at the Department of Oral Medicine, Dental School, Mashhad University of Medical Sciences, Mashhad, Iran, during the period between September 2004 and September 2008 were reviewed for cases of RH. Records with both clinical and histopathological diagnoses of RHs were selected. Data including age and sex, as well as the anatomical location and the clinical characteristics of the lesion, such as base, surface, size, and color were collected.

As for the etiology and the histopathologic differences, RHs was classified as: trumatic fibroma (TF) (Figure 1a), pyogenic granuloma (PG) (Figure 1b), peripheral giant cell granuloma (PGCG) (Figure 1c), peripheral fibroma with calcification (PFC) (Figure 1d), and denture hyperplasia (Figure 1e).


Figure 1. (a) Traumatic fibroma in the buccal mucosa of a 34-year-old male with ulcerated surface. (b) Pyogenic granuloma in the hard palate of a 16-year-old boy due to factitious trauma. (c) A saddle-shaped peripheral giant cell granuloma. (d) A large-sized peripheral fibroma with calcification and tooth displacement. (e) Denture hyperplasia in a 76-year-old male.

**a:**
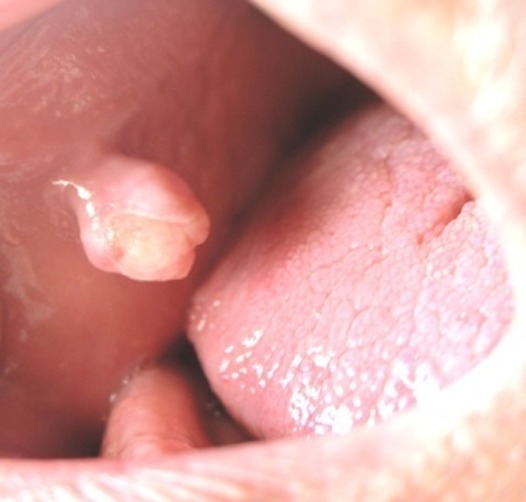

**b:**
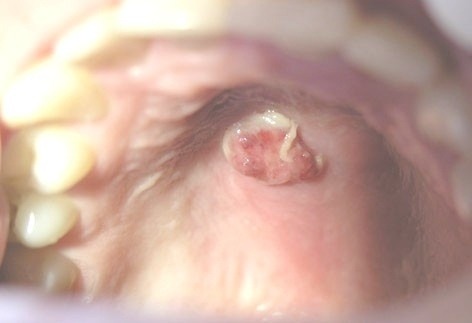

**c:**
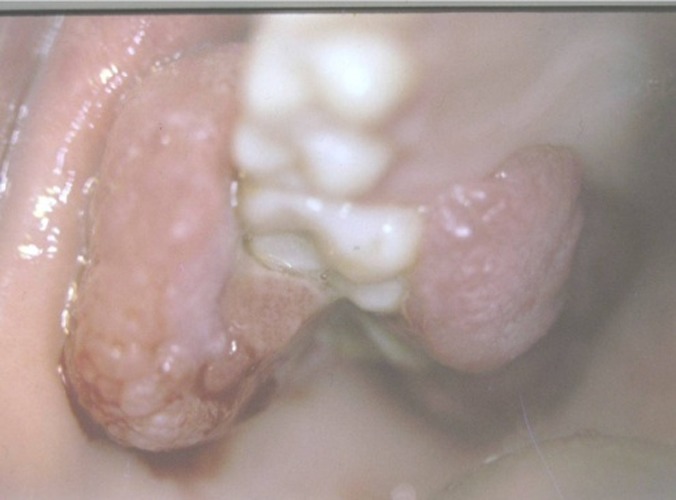

**d:**
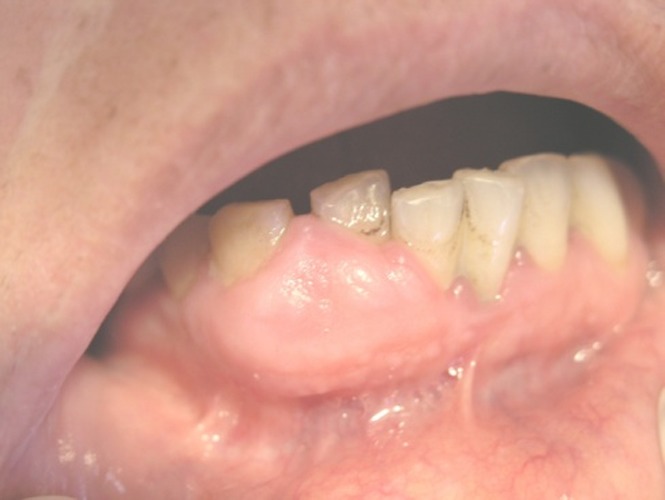

**e:**
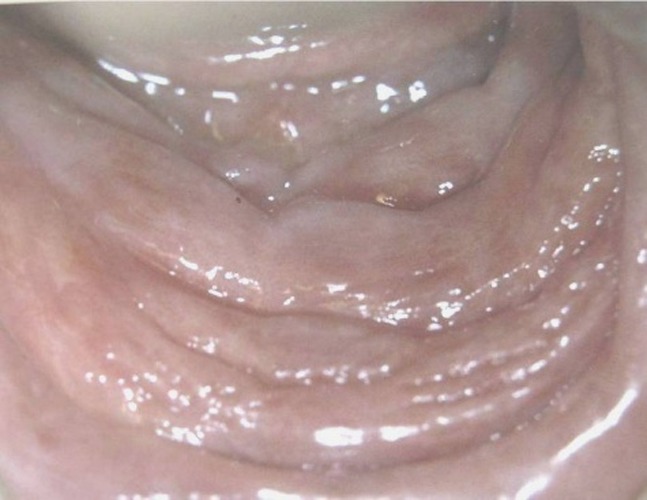

Data were analyzed using SPSS 13.5 software (Michigan, US). Descriptive statistics was employed to report the findings. Chi-square test was used for evaluation of differences in frequencies among groups. Statistical significance was set as p<0.05.

## Results

From a total of 123 RH cases diagnosed during the assessed period, TF was the most common lesion (50 cases, 40.6%) followed by PGCG (34 cases, 27.6 %), PG (31 cases, 25.2%), PFC (7 cases, 5.69%) and hyperplasia caused by denture (1 case, 0.81%) (Table 1).

**Table 1:** Distribution of patients with reactive hyperplasia according to age, sex and site of involvement (n = 123)

	Sex		Mean	Site						
Lesion	Male	Female	age	Palate	Tongue	Gingiva	Lip	Vestibule	Buccal mucosa	Total
Traumatic fibroma (TF)	14	36	43.30	4	14	10	5	2	15	50
Pyogenic granuloma (PG)	12	19	35.03	3	1	20	2	2	3	31
Peripheral giant cell granuloma (PGCG)	14	20	34.44	1	0	29	0	3	1	34
Peripheral fibroma with calcification (PFC)	1	6	44.17	1	0	5	0	0	1	7
Denture hyperplasia	1	0	56	0	0	0	0	1	0	1
Total	42	81	38.8	9	15	64	7	8	20	123

### Age and Sex

Table 1 shows the incidence of various types of RH according to age and sex. There were 81 females (mean age, 40.06 years) and 42 male patients (mean age, 36.77 years), with a male to female ratio of 1:1.9. Most of the lesions were more common in women, including PG (p=0.209), TF (p=0.02), PGCG (p=0.303), and PFC (p=0.059), while denture hyperplasia (one case) was observed in a male patient.

Mean age of the patients in this study was 38.88 ± 18.50 years. A higher degree of occurrence was observed in the third and the forth decades of life regarding lesions such as PG (38.7%) and PGCG (38.23%). TF (44%), PFC (57.4%) and denture hyperplasia (100%) were common in the fifth and the sixth decades of life.

### Site

TF was most commonly seen in the buccal mucosa (15/50, p=0.03) and on the tongue (14/50, p=0.03). Other intraoral sites of TF included gingiva, lip, palate and vestibule. In the present study, gingiva was the most common location of PG (20/31, p=0.00), PGCG (29/34, p=0.00), and PFC (5/7, p=0.102). A significant relationship between the site and the type of the lesion was only observed in PG and PGCG affecting gingiva. Overall, 64 RH cases involved the gingiva. The only case of denture hyperplasia was in the vestibule of maxilla. Table 1 shows the location of these reactive lesions.

### Clinical Feature

Chief complaint in the majority of cases was swelling (76.2%). Other complaints included pain (5.7%), ulceration (3.3%) and a burning sensation (8%). Some patients had more than one of the complaints. The lesions ranged from 8 mm to 6 cm in size. The largest diameter in most of the TF cases in the present study was 1 cm. The duration of the lesions at the time of diagnosis ranged from 1 to 12 months.

79.8 % of lesions were polypoid and the lesions had a sessile base in 69% of the cases (Table 2). Most of TF (66%), PG (38.7%), GCG (50%), and PFC (62.5%) were firm (Table 2). The surface of most lesions (91.1%) was smooth. Other lesions had an ulcerated (4.8%) or a rough (4.1%) surface.

**Table 2:** Distribution of reactive hyperplastic (n = 123) and polypoid (n = 99) lesions according to base and consistency of the lesion

	Base of polypoid lesions			Consistency			
Lesions	Sessile	Pedunculated	Total	Firm	Rubbery	Soft	Total
Traumatic fibroma (TF)	22	13	35	33	11	6	50
Peripheral giant cell granuloma (PGCG)	25	5	30	17	9	8	34
Pyogenic granuloma (PG)	16	10	26	12	8	11	31
Peripheral fibroma with calcification (PFC)	6	1	7	4	2	1	7
Denture hyperplasia	1	0	0	0	1	0	1
Total	70	29	99	66	31	26	123

### Treatment and Follow-up

All lesions were surgically excised and sent for histopathological examination. The cause of all the lesions was identified as local irritation, and the source of trauma was eliminated in all cases. Due to retrospective design of the study, the follow-up appointment trends were not similar and no comparison could be performed.

## Discussion

Reactive hyperplasia is a benign lesion caused by local and chronic trauma. There are a few reports on the prevalence, clinical manifestations, and histopathologic pattern of these lesions in Iran.^4 , 5^ Mashhad University of Medical Sciences is the main referral center for the patients in the northeast of the country, including North Khorasan, Razavi Khorasan and South Khorasan provinces. This study only included RH cases with a histopathological diagnosis. There were, for example, many cases of denture hyperplasia among evaluated records; however, as for the absence of histopathplogical data, they had to be excluded from the study.

Age of onset is an important clinical parameter when the differential diagnosis of a lesion is being formulated. In our study, the age distribution of patients with different types of RHs was similar to figures reported in other countries.^6 , 7^ In one study in Kerman province, central Iran, Zarei et al^4^ studied 172 cases of RH and found the mean age of the patients to be 37 years. Mean age in our study was higher than some other reports. Giansanti & Waldron^8^ reviewed 720 cases of peripheral giant cell granuloma and reported a mean age of 30 years. Of 123 cases studied in the present study, 34.1% were seen in men and 65.9% in women, which is in agreement with previous literature.,^6 , 9^ However, female predominance in PGCG (F:M=20:14) in the present study was inconsistent with a previous report of male predilection (M:F=1.4:1).

We found that the principal site affected by PG was gingiva. This finding is consistent with those of other studies.,^10^ Also in line with other reports,,^11^ the most common site of involvement was gingiva in most RHs. In our study, only 4.8% of lesions were ulcerated which is lower than another report (39.5%).^4^Similar with other reports, smooth surface and sessile base was the typical clinical feature in RH. ^1, 12 , 13^ However, it has been shown, in one report, that most of PGCGs were pedunculated.^3^ This difference can be due the lack of a unique terminology among scientists. Generally, prognosis of RHs is good unless the source of trauma persists. In this case frequent recurrence is possible.

## Conclusion

In summary, the features of reactive hyperplasia among our patients were similar to those reported previously, although there are some differences due to demographic variations. It seems that a unique terminology should be defined so that all clinical descriptive analyses can be compared.
